# OA01 Fragile bones, complex lives: osteogenesis imperfecta as a paradigm for metabolic bone disease in adults

**DOI:** 10.1093/rap/rkag113.001

**Published:** 2026-11-03

**Authors:** Kirishananth Rajaseelan, Arjun Narayana, Anupama Nandagudi

**Affiliations:** Mid and South Essex NHS Foundation Trust, Essex, United Kingdom; Mid and South Essex NHS Foundation Trust, Essex, United Kingdom; Mid and South Essex NHS Foundation Trust, Essex, United Kingdom

## Abstract

**Introduction:**

Osteogenesis Imperfecta (OI) is a heritable connective tissue disorder caused by defective collagen synthesis, phenotypically manifesting as skeletal fragility, recurrent fractures and progressive disability. Approximately 5,000 individuals in the UK are affected yet OI remains incompletely managed in adulthood. We present four patients with OI, ages spanning more than eight decades. We illustrate the complexity of metabolic bone disease assessment and management, including: serial bone-density surveillance, calcium and mineral metabolism disturbance, pharmacotherapy, co-existence of parathyroid disease, and multidisciplinary integration by NHS Eastern Rare Bone Disease Network.

**Case description:**

**Patient 1 (Type I)** presented with persistent cervical and thoracic spinal pain and multiple fragility fractures. Bone profile remained normal throughout. DEXA demonstrated lumbar improvement on strontium ranelate but deterioration in the left forearm. Sequential therapy included alendronic acid, teriparatide, strontium ranelate, and denosumab. Despite treatment, functional decline progressed from independence (2013) to wheelchair dependence (2018).

**Patient 2 (Type IV)** experienced mechanical back and neck pain with recurrent low-impact fractures. Bone density remained stable (T-score −2.7 to −2.0) over 14 years. The ERBON-MDT recommended bone-turnover markers (CTx, P1NP) to guide teriparatide use. She was treated with pamidronate, then risedronate for 10 years, followed by a bisphosphonate holiday during which a fibular fracture occurred, prompting therapy restart.

**Patient 3** developed hypercalcaemia and hyperparathyroidism on teriparatide, requiring parathyroidectomy. Subsequent ibandronic acid, zoledronic acid, and denosumab improved bone density but caused rebound pain and immobility. Re-introduction of zoledronic acid restored function; discontinuation led to bilateral femoral fractures.

**Patient 4** had recurrent childhood fractures misidentified as non-accidental injury, vitamin D deficiency, and raised ALP. DEXA was limited by prosthetic artefacts. Risedronate was continued after intolerance to alendronic acid. Wheelchair dependence and depression developed secondary to immobility.

**Discussion:**

Our case series illustrates complexity in OI management in adults: pharmacological decision-making is constrained by inadequate evidence and compounding comorbidity.

**Bisphosphonate duration dilemma** Management of Patient 2 exemplifies the unresolved challenge of bisphosphonate duration in adult OI. With 10-years of risedronate, the optimal duration for treatment was unknown, corroborated in literature highlighting no evidence basis to guide therapy duration. Unlike postmenopausal osteoporosis, where drug holiday protocols are established from trials (FLEX and HORIZON), no equivalence exists for OI. A drug holiday commenced in Feb-2020 resulted in left fibular fracture and recommencement in September-2021 illustrates the risk of cessation without clear evidence-based guidance.

**Comorbidities and multidisciplinary approach** Patient  3’s course illustrates the interplay between OI and endocrine dysfunction. Hypercalcaemia and hyperparathyroidism complicated teriparatide use, necessitating parathyroidectomy. Rebound pain on denosumab withdrawal and subsequent bilateral femoral fractures highlight the risks of abrupt cessation and need for transition strategies between therapies. Patient 4’s history of misdiagnosed childhood fractures and depression secondary to immobility underscores psychosocial burden of chronic bone disease. Functional limitations and mental health sequelae require integrated multidisciplinary support.

**DEXA limitations** Prosthetic artefacts prevented hip assessments in patients 1 and 4 in monitoring phases. Degenerative changes artefactually elevated lumbar readings in patients 3 and 4. Paradoxically, Patient 2’s DEXA demonstrated lumbar improvement during drug holiday however sustained left fibula fracture, illustrating density monitoring may inadequately identify fracture risk in OI – pathologically fragility driven by collagen composition over BMD.

**Specialist Network Input** The ERBON-MDT recommendation to use bone-turnover markers (CTx, P1NP) to guide teriparatide initiation reflects growing role of biochemical surveillance in precision therapy. Deriving intricate plans require broader mechanistic thinking, difficult to achieve with a standard rheumatological review. Further, access to OI remains variable across UK, hence necessitating such networks to coherently improve management strategies and ultimately prognosis and QoL.

**Key learning points:**

1. **Screening beyond Bones:** Fundamentally OI is a systemic connective disorder, with varying degrees of phenotypic manifestations in other human systems. Therefore, significant importance to screen other symptoms and to manage beyond bone changes.

2. **Bisphosphonates exit strategy at initiation –** Bisphosphonates are started primarily to improve bone characteristics. Upon initiation, clinicians should holistically establish criteria for pausing therapy and plan for monitoring in drug holiday window in prospect of retreatment. However, the evidence-base is sparse for guiding point of cessation, warranting further trial data to inform guidelines and clinicians on optimal duration.

3. **Denosumab discontinuation strategy:** Rebound-associated fractures occur in ∼1 in 14 patients without sequential antiresorptive therapy. This risk is compounded in adult OI due to collagen composition insufficiency. A transitional agent could be warranted to reduce pain manifestations and further fracture risks, especially when intending completely withdraw.

4. **Supplementing DEXA monitoring:** Artefacts due to prosthetics or degenerative OA and discordance between T-Score and fracture risk in OI limit the interpretation of DEXA results in monitoring. Bone turnover markers may prove effective if integrated to routine monitoring, though its cost-effectiveness needs establishment.

5. **Specialist MDT discussions:** Multidisciplinary networks enable comprehensive evaluation of complex OI cases, integrating bone metabolism, pharmacology, and comorbidities. Such collaboration fosters individualized management plans and supports evidence development for rare bone disease care.

